# Domain Knowledge Constrained Symbolic Regression for Optimising Dermal Drug Formulations

**DOI:** 10.1007/s11095-026-04170-9

**Published:** 2026-08-03

**Authors:** Yu Zhang, Xilu Wang, Dimitrios Tsaoulidis, Tao Chen

**Affiliations:** 1 School of Chemistry and Chemical Engineering, University of Surrey, Guildford, GU2 7XH UK; 2 School of Computer Science and Electronic Engineering, University of Surrey, Guildford, GU2 7XH UK

**Keywords:** dermal delivery, domain knowledge constrained symbolic regression, formulation optimisation, genetic programming, interpretable model discovery

## Abstract

**Purpose:**

Interpretable dynamic models that remain physically admissible under extrapolation are essential for dermal formulation optimisation. Mechanistic models are often labour-intensive to derive, whereas unconstrained data-driven models may violate physical constraints and degrade beyond observed conditions.

**Methods:**

We developed a domain knowledge constrained symbolic regression (DKC-SR) framework to discover compact, interpretable dynamic models for cumulative release directly from time-series experiments. Domain knowledge was embedded through a restricted operator set and feasibility constraints over the bounded optimisation domain. Candidate expressions were evaluated by numerically solving the dynamic differential equations and selected using an information-criterion trade-off across a level-wise set of complexity-controlled models. The final surrogate was coupled with the covariance matrix adaptation evolution strategy for formulation optimisation.

**Results:**

In an ibuprofen formulation case study, the selected model provided a formulation ranking that, combined with experimental validation, guided the optimiser to a narrow high-performing region; a representative optimum was experimentally confirmed to exceed the historical best. Although the surrogate was more reliable for ranking and optimisation guidance than for exact quantitative calibration, it remained physically admissible and numerically stable across the design space.

**Conclusions:**

DKC-SR provides a compact, interpretable, and optimisation-ready route to modelling dermal release under bounded design constraints.

**Supplementary Information:**

The online version contains supplementary material available at https://doi.org/10.1007/s11095-026-04170-9.

## Introduction

Topical drug formulations play a central role in pharmaceutical development, enabling localised delivery with reduced systemic exposure and improved patient compliance [1, 2]. A key determinant of their efficacy is dermal permeation, which governs both the rate and extent of active pharmaceutical ingredient (API) delivery into and across the skin [3]. The *in vitro* release test (IVRT) is a well-established methodology for characterising formulation release behaviour. It provides time-series measurements of cumulative API release under controlled conditions, typically using synthetic membranes to separate the donor and receptor compartments in a Franz diffusion cell [4–7]. Such measurements form an empirical basis for understanding release behaviour and for developing predictive models that can ultimately guide formulation design and optimisation.

Modelling approaches for dermal release can be broadly grouped into mechanistic transport models and data-driven surrogates. Mechanistic transport models are typically formulated from mass conservation with Fickian diffusion flux (e.g., diffusion-partition models) and are interpretable, with parameters that can in principle transfer across related systems [8]; related dermal transport problems have also been addressed via end-to-end computational workflows that parameterise physiologically based pharmacokinetic (PBPK)-based percutaneous absorption models [9]. However, they often depend on simplifying assumptions, are labour-intensive to derive and calibrate, and may be difficult to generalise across multiple excipients and wide formulation ranges [10, 11]. In parallel, empirical/quantitative structure–property relationship (QSPR) correlations (e.g., Potts-Guy-type relationships for permeability estimation [12, 13]) provide lightweight data-driven predictions within their calibration envelope, but they are not designed to capture formulation-dependent release dynamics. In contrast, machine learning (ML) approaches can flexibly capture nonlinear relationships from experimental datasets, yet they frequently operate as statistical “black boxes” with limited interpretability [14, 15].

While mechanistic transport models and data-driven surrogates each offer distinct advantages, neither alone typically provides the balance of predictive performance, interpretability and domain-wide reliability needed for formulation design. This motivates the use of data-driven surrogates with domain knowledge constraints, so that model evaluations remain meaningful and recommendations are experimentally actionable. At the same time, selecting empirical equations from a pre-defined pool can be restrictive, as pre-specified forms may lack the flexibility to capture formulation-dependent dynamics beyond the assumptions of classical transport relations. A more promising direction is therefore to learn compact rate expressions directly from time-series data, in a form that shares structural features with first principles models and supports transfer to analogous systems governed by similar mechanisms [16, 17].

Symbolic regression (SR) has emerged as a promising paradigm for narrowing the gap between mechanistic and purely data-driven prediction by directly discovering closed-form expressions from data [18]. In contrast to many statistical and ML regressors [19] that yield data-driven surrogates, SR searches over functional forms and returns explicit mathematical expressions that map inputs to outputs. This property makes SR inherently interpretable and, in favourable cases, capable of recovering governing relationships that offer mechanistic insight while maintaining competitive predictive accuracy [20]. Accordingly, SR has gained traction across a wide range of scientific and engineering problems, including biological systems [21], physics-informed discovery tasks [22], and chemical engineering applications where compact models are valued for analysis and design [17]. Unconstrained SR, however, can also yield physically implausible or numerically brittle expressions, which is especially problematic when the model is deployed as an optimisation surrogate.

Symbolic regression methods range from evolutionary approaches, most notably genetic programming (GP), which evolves expression trees over a chosen operator set [18, 23–25], to library-guided variants such as ALAMO (Automated Learning of Algebraic Models for Optimisation) [26] and SINDy (Sparse Identification of Nonlinear Dynamics) [27], and physics-guided variants such as AI-Feynman [28]. We adopt tree-based GP because, unlike library-based methods, it requires no predefined candidate library and can naturally incorporate domain-specific operators and hard constraints into the search. These properties are essential for the constrained, optimisation-oriented discovery pursued here. Domain knowledge guided and transfer-learning methods have also been applied in related data-driven modelling, for example to soft sensing under imperfect measurements [29] and sensor-based prognosis and condition-monitoring tasks [30], typically transferring learning representations to improve data efficiency. In contrast, we embed physical and numerical feasibility directly into symbolic-equation discovery, so that the resulting interpretable rate law remains mechanistically admissible and safely evaluable as an optimisation surrogate.

Despite its appeal, symbolic regression may not be directly applicable to topical formulation problems. The unconstrained search space readily produces expressions that are physically inadmissible (e.g., negative rates) or numerically ill-posed when evaluated across a bounded formulation domain, leading to undefined operations, overflow, or singularities. These failures are amplified in optimisation, where the optimiser repeatedly probes boundary regions and extrapolates beyond the training data. We therefore develop a domain knowledge constrained symbolic regression (DKC-SR) framework that embeds domain priors through a restricted operator set and hard feasibility screening, ensuring every candidate rate law remains physically meaningful and safely evaluable across the entire design space. Candidate expressions are evaluated by numerically solving the induced ordinary differential equations (ODEs), and a level-wise set of best models is retained across complexities before selecting a parsimonious final surrogate via an information-criterion trade-off. The selected surrogate is then coupled with the covariance matrix adaptation evolution strategy (CMA-ES) [31–33], to maximise the final-time cumulative API release under bounded excipient constraints in formulation optimisation. This evaluates whether the discovered model provides stable and physically admissible guidance within the formulation design space.

Although demonstrated here on IVRT time-series for topical formulations, the DKC-SR framework is readily extensible to other dermal delivery settings and related transport problems, including longer horizons and protocols that expose richer mechanistic signatures. It is worth separating two kinds of contribution. Methodologically, our contribution is a domain-tailored constraint strategy oriented towards downstream optimisation. Unlike library-based constrained methods (e.g., SINDy) or generic shape-constrained symbolic regression, and unlike constraint-guided kinetic-model discovery [34] that targets data efficiency, we enforce hard constraints over the entire bounded design space that the optimiser will subsequently probe, promoting safe evaluability everywhere the search may go. The constraints are combined with a positivity-preserving operator (softplus) and full-variable-coverage screening so that the discovered ODE remains physically admissible, numerically stable and composition-dependent throughout optimisation. In terms of application, to our knowledge this is the first use of domain knowledge constrained symbolic regression for dermal release dynamics coupled to formulation optimisation.

## Experimental Setting

Ibuprofen-loaded poloxamer 407-based formulations were used as the experimental system in this study. The overall formulation composition followed our previous protocols [35–37], comprising ibuprofen, medium chain triglycerides, poloxamer 407, ethanol, propylene glycol (PG), and ultra-pure water. Three excipients were treated as optimisable formulation variables, i.e., poloxamer 407 (${x}_{1}$), ethanol (${x}_{2}$), and PG (${x}_{3}$), with concentration bounds defined in the design space (in Table 1), while the remaining components were kept fixed.

**Table 1:** Design space of key excipients considered in ibuprofen formulations

Excipients	Range of values (%w/w)
Poloxamer 407 (${x}_{1}$)	20—30
Ethanol (${x}_{2}$)	10—20
Propylene glycol (${x}_{3}$)	10—20

IVRT experiments were conducted using vertical Franz diffusion cells with synthetic nylon membranes separating the donor and receptor compartments [4, 38]. Phosphate buffered saline (PBS) was used as the receptor medium. After membrane mounting, a 300 mg aliquot of formulation was applied uniformly on the membrane surface, and the donor chamber was sealed with parafilm to minimise ethanol evaporation. Receptor samples (1 ml) were withdrawn at 0.5, 1, 2, 3, 4, 5, and 6 h, with PBS replenishment after each sampling, and sink conditions were maintained throughout the experiments.

Ibuprofen concentration in receptor samples was quantified by UV–vis spectrophotometry over the 200—300 nm range [39]. Calibration standards were prepared by serial dilution from a 500 $\upmu \mathrm{g}/\mathrm{m}\mathrm{l}$ ibuprofen stock solution in PBS (0.5—500 $\upmu \mathrm{g}/\mathrm{m}\mathrm{l}$). To mitigate nonlinear absorbance responses at higher concentrations, partial least squares (PLS) regression was used for multivariate calibration based on selected informative wavelengths, following established spectroscopic calibration practices [40, 41]. The resulting concentration time series was converted into cumulative release per unit membrane area. Replicate IVRT runs were performed for each formulation. For model fitting, replicate measurements were averaged at each sampling time to obtain a single mean release trajectory per formulation and reduce measurement noise, yielding the time-series dataset used for dynamic model discovery and formulation optimisation. In addition, experimental variability was retained separately for validation experiments but was not explicitly propagated into the SR loss function in this study.

## Methodology

**Fig. 1:**
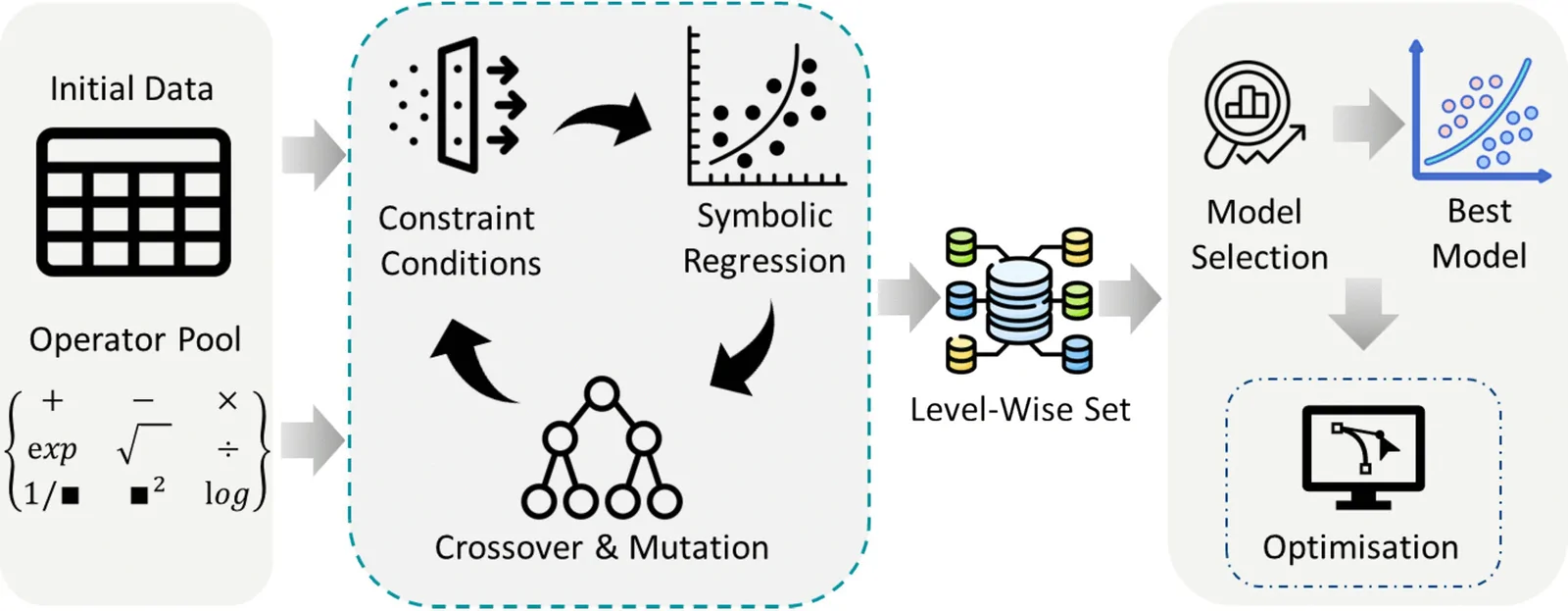
Conceptual overview of the domain knowledge constrained symbolic regression and optimisation framework.

This section outlines an overview of the domain knowledge constrained symbolic regression and optimisation framework proposed for optimising dermal drug formulations in Fig. 1. Starting from an initial experimental design, IVRT experiments generate time series release profiles for a set of formulations defined by excipient concentrations. Based on the collected training data, an SR model is trained by using a GP-based algorithm for providing interpretable dynamic models, guided by domain knowledge derived from exact physical expressions. Specifically, prior domain knowledge is embedded through a restricted operator set and structural constraints derived from physical relations. The algorithm optimises candidate expressions by minimising the residual loss between experimental measurements and the model’s predictions until termination criteria are met (the maximum number of iterations is reached or convergence). From a generated level-wise set of expressions, the best model is selected using the Akaike Information Criterion (AIC). Finally, the discovered dynamic model is embedded into a global optimisation routine to guide formulation optimisation and is validated experimentally.

### Problem Setting for ODE-based Dynamic Model Discovery

The framework operates directly on time-series experimental data, which serve as the basis for discovering closed-form rate expressions. Let $\mathbf{x}=\left\{{x}_{1},{x}_{2},\dots ,{x}_{d}\right\}\in {\mathbb{R}}^{d}$ denote the set of explanatory variables representing experimental factors, and $t\in {\mathbb{R}}^{+}$ denote the temporal index. The observed response is denoted by $Q\left(t\right)\in {\mathbb{R}}$, measured at discrete time points $\left\{{t}_{1},{t}_{2}, \dots ,{t}_{n}\right\}$. Thus, the resulting dataset can be formally represented as:

1 $$\mathcal{D}=\{t,\mathbf{x},Q\left(t\right)\}$$

where $\mathbf{x}$ denotes the concentration of the formulation excipients, $Q\left(t\right)$ is the cumulative release at time ${t}_{j}$. For numerical stability, the response Q was normalised to a dimensionless state $\widetilde{Q}\left(t\right)\in [\mathrm{0,1}]$ prior to symbolic regression and ODE integration. Specifically, $\widetilde{Q}\left(t\right)=\frac{Q\left(t\right)-{Q}_{min}}{{Q}_{max}-{Q}_{min}}$, where we set ${Q}_{min}=0$ by definition because cumulative release satisfies $Q\left(0\right)=0$. ${Q}_{max}$ was computed from the training set ($Q_{max}=3008.20\;\mu g/cm^2$ in this study) and then fixed for all test and optimisation evaluations. Analogously, each formulation variable was likewise min–max scaled from its design range in Table 1 to the unit interval [0,1] before entering the symbolic rate expressions (e.g., poloxamer 407 from 20–30% to 0–1). In the rate expressions, ${x}_{1},{x}_{2}, \mathrm{a}\mathrm{n}\mathrm{d}\text{ } {x}_{3}$ therefore denote these scaled inputs, whereas Table 1 and the reported formulations use raw % w/w values. All model discovery and optimisation were performed in the $\widetilde{Q}$ space; predictions were then inverse-transformed to raw ($\upmu \mathrm{g}/{\mathrm{cm}}^{2}$ in this study) for reporting.

The modelling objective is to discover an explicit functional relation,

2 $$d\widetilde{Q}/dt\approx f\left(\widetilde{Q},\mathbf{x};\theta \right)$$

where $f\left(\bullet \right)$ is a closed-form expression obtained through SR, and θ represents the fitted constants. This formalisation enables the symbolic model to jointly capture the effects of both experimental factors and temporal dynamics, thereby aligning with the physical requirements of the system (dermal release dynamics) under study. Next, we describe each component of the proposed methodology in detail.

### Domain Knowledge Constrained Symbolic Regression

Symbolic regression seeks to discover explicit analytical expressions that describe the underlying dynamics of dermal drug release. Given a training set of labelled data, the goal in SR is to find an interpretable expression, where the underlying function generalises well beyond the training data [25]. In this study, SR is implemented using a GP framework [23, 42, 43], designed to evolve interpretable differential equations that relate formulation variables $\mathbf{x}$ and cumulative release amount $Q\left(\mathrm{t}\right)$.

Here, "domain knowledge constrained" means translating established physical and numerical knowledge of dermal release into explicit restrictions on the symbolic search, so that candidate rate laws remain physically meaningful and safely evaluable across the design space. This knowledge is embedded through the restricted operator set (Section "Operator Set") and the hard feasibility constraints (Section "Hard Constraints for Feasibility Screening"). It comprises the non-negativity and monotonicity of cumulative release, the diffusion/dissolution rate-law forms it follows, its dependence on the full excipient composition, and the numerical admissibility required of an ODE surrogate queried repeatedly during optimisation.

#### Operator Set

The operator set is restricted to mathematically admissible primitives that commonly appear in diffusion-based and dissolution-based rate laws [8, 11], namely:

3 $$\mathcal{O}=\left\{+,-,\times ,\div ,\mathrm{pow}\left(\bullet \right),\mathrm{log}\left(\bullet \right),\mathrm{exp}\left(\bullet \right),softplus\left(\bullet \right)\right\}$$

The inclusion of these operators is guided by well-established release models such as:

i) Higuchi model: involving square root time scaling and linear combinations [44, 45];
ii) Korsmeyer–Peppas/Peppas–Sahlin models: involving power-law behaviour and exponential-like contributions [46–48];
iii) Fickian diffusion models: involving logarithmic and exponential terms from analytical solutions to diffusion equations [49];

Nonphysical operators such as trigonometric or discontinuous functions are intentionally excluded because they lack mechanistic interpretation for cumulative release and often lead to non-physical release dynamics (e.g., negative rates or non-monotone cumulative release).

The softplus function,

4 $$softplus\left(z\right)=In\left(1+e^z\right)$$

is incorporated for two reasons. Firstly, the release rate $d\widetilde{Q}/dt$ must remain non-negative for all t and all admissible x. Softplus acts as a smooth approximation to ${max}\left(\mathcal z,0\right)$, ensuring $d\widetilde{Q}/dt\ge 0$ without introducing nondifferentiable points that can complicate ODE integration. Secondly, it enables smooth enforcement of the non-negativity constraint in a manner consistent with physical release dynamics. Since diffusion and dissolution dynamics yield non-negative flux terms (e.g., in Fick’s law or dissolution-controlled release), softplus prevents the GP from generating negative fluxes, thereby embedding physical realism directly through the operator choice. Such smooth positivity-enforcing operators have also been explored in physics-informed machine learning for dynamic modelling [50].

#### Hard Constraints for Feasibility Screening

The symbolic search space contains many mathematically valid but physically inadmissible expressions. We therefore apply hard constraints so that only rate laws that are physically admissible and numerically safe over the entire bounded design space are evaluated. Four requirements are enforced.

Physical consistency. Because the release rate (flux) is non-negative, cumulative release is non-decreasing [11, 38, 45]. The rate expression must therefore satisfy:

5 $$d\widetilde{Q}/dt\ge 0$$

consistent with irreversible donor-to-receptor diffusion and standard IVRT guidance.

Full variable coverage. To avoid degenerate, composition-independent solutions, every formulation variable must appear explicitly in the expression:

6 $$\left\{\widetilde{Q},{x}_{1},{x}_{2},\dots ,{x}_{d}\right\}\subseteq \mathrm{V}\mathrm{a}\mathrm{r}\mathrm{s}\left({f}_{\theta }\right)$$

where $\mathrm{V}\mathrm{a}\mathrm{r}\mathrm{s}\left({f}_{\theta }\right)$ denotes the set of variables present in the expression. This requirement ensures that the model captures the mechanistic influence of excipient concentrations on release dynamics and prevents the regression from collapsing into a partially informative but physically incomplete representation (e.g., dropping a formulation variable simply because its effect is weak).

Structural and numerical admissibility. Operations prone to instability are disallowed, in particular placing the state variable in denominators or constructing excessively nested exponential forms. Representative restrictions can be expressed as:

7 $$\widetilde{Q}\notin Denominator\left({f}_{\theta }\right), neste{d}_{\mathrm{exp}\left({f}_{\theta }\right)}\le 1$$

and expression tree depth is bounded to keep the rate laws compact and intelligible,

8 $$\mathrm{d}\mathrm{e}\mathrm{p}\mathrm{t}\mathrm{h}\left({f}_{\theta }\right)\le {D}_{\mathrm{m}\mathrm{a}\mathrm{x}}$$

Optimisation-domain validity. Since the discovered model will subsequently be used for global optimisation of formulation compositions, candidate expressions must remain well-defined throughout the feasible ranges of all formulation variables. Any functional form that becomes undefined within the optimisation ranges ${x}_{k}\in \left[{a}_{k},{b}_{k}\right]$ is excluded. This ensures that the resulting dynamic model does not introduce artificial discontinuities or singular barriers during optimisation.

Collectively, these constraints define the principles that guide the SR search. Their specific application within the GP algorithm is described in Section "GP-based Model Generation and Evaluation".

#### GP-based Model Generation and Evaluation

**Fig. 2:**
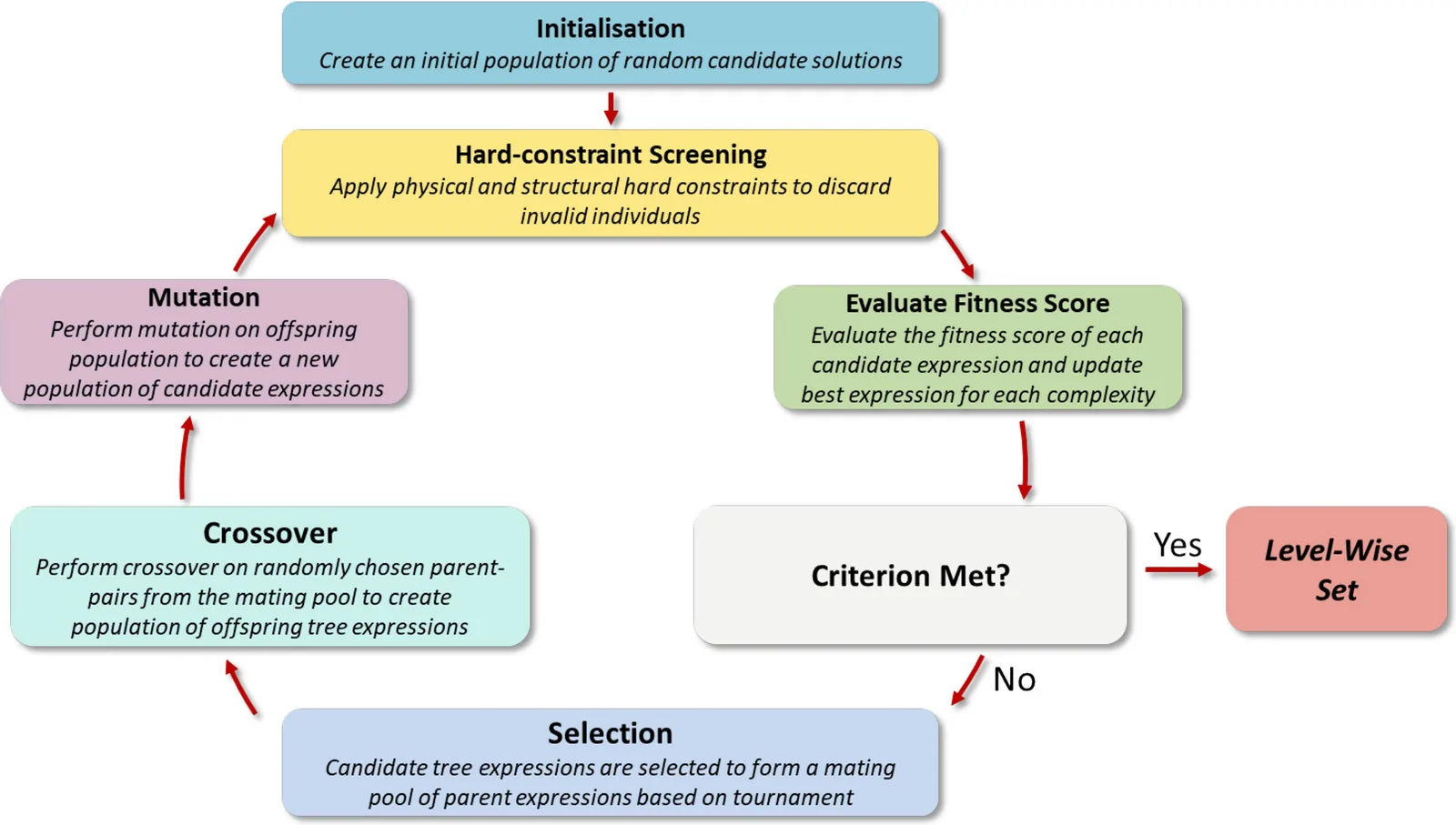
The flowchart of the domain knowledge constrained GP-based symbolic regression.

The discovery of rate expressions is performed using a GP framework specifically adapted to operate under the constraints defined earlier. The evolutionary loop illustrated in Fig. 2 is designed such that constraint enforcement is embedded directly into the GP search dynamics. This ensures that only feasible expressions, both physically meaningful and numerically well-posed, are ever evaluated by the fitness function.

The algorithm begins by generating an initial population ${\mathcal{P}}_{0}={\left\{{f}_{\theta }^{i}\right\}}_{i=1}^{{N}_{p}}$ of random tree-structured expressions using the operator set $\mathcal{O}$, where ${N}_{p}$ is the population size. Each newly generated individual is immediately subjected to hard constraint screening, ensuring that only feasible/admissible expressions enter the evolutionary loop. This creates a strictly filtered search space in which all surviving individuals are physically and numerically viable, thereby improving both convergence and robustness.

Specifically, an individual ${f}_{\theta }$ is discarded if it violates any of the following conditions:

(i) non-negativity of flux, ${f}_{\theta }\left(\widetilde{Q}, \mathbf{x}\right)\ge 0$ for all sampled states;
(ii) full variable coverage, $\left\{\widetilde{Q},{x}_{1},{x}_{2},\dots ,{x}_{d}\right\}\subseteq \mathrm{V}\mathrm{a}\mathrm{r}\mathrm{s}\left({f}_{\theta }\right)$;
(iii) structural admissibility, which excludes forbidden patterns such as $\widetilde{Q}$ in denominators, invalid logarithmic arguments, or excessively nested exponentials; and
(iv) formulation-domain validity, meaning that ${f}_{\theta }$ is safely evaluable on the entire experimental domain without numerical overflow or undefined operations.

Individuals that pass the hard constraints are evaluated using a fitness function that combines data fidelity with soft regularisation terms. The data misfit is defined as

9 $${\mathcal{L}}_{data}\left({f}_{\theta }\right)=\sum\nolimits_{i=1}^{N}\sum\nolimits_{j=1}^{n}{\left({\widetilde{Q}}_{sr}^{\left(i\right)}\left({t}_{j};{f}_{\theta }\right)-{\widetilde{Q}}_{\mathrm{o}\mathrm{b}\mathrm{s}}^{\left(i\right)}\left({t}_{j}\right)\right)}^{2}$$

where ${\widetilde{Q}}_{\mathrm{o}\mathrm{b}\mathrm{s}}^{\left(i\right)}\left({t}_{j}\right)$ denotes the experimentally measured cumulative release for the formulation i at time ${t}_{j}$, and ${\widetilde{Q}}_{sr}^{\left(i\right)}\left({t}_{j};{f}_{\theta }\right)$ is obtained by numerically solving the ODE

10 $$d\widetilde{Q}/dt={f}_{\theta }\left(\widetilde{Q},{\mathbf{x}}^{\left(i\right)}\right), \widetilde{Q}\left(0\right)=0$$

using a fixed-step fourth-order Runge–Kutta scheme with automatic step subdivision to ensure stability and accuracy [51].

To discourage overly complex expressions that may overfit training data while sacrificing interpretability, a complexity penalty is incorporated into the fitness function. This complexity penalty is computed as the weighted sum of the operator nodes in the expression tree (terminals excluded),

11 $${\mathcal{L}}_{comp}\left({f}_{\theta }\right)=\sum\nolimits_{n\in \mathrm{n}\mathrm{o}\mathrm{d}\mathrm{e}\mathrm{s}\left({f}_{\theta }\right)}\omega \left(n\right)$$

where $\omega \left(n\right)=1$ for binary operators ($+,-,\times ,\div$) and unary functions (e.g., log, exp, pow). This definition follows the complexity metric commonly used in SR [24]. In this work, the structural complexity level was defined as $\mu ={\mathcal{L}}_{comp}$, i.e., the weighted node-count complexity in Eq. (11).

The overall fitness score minimised during evolution is therefore

12 $$\mathcal{F}\left({f}_{\theta }\right)={\mathcal{L}}_{data}\left({f}_{\theta }\right)+{\lambda}_{comp}{\mathcal{L}}_{comp}\left({f}_{\theta }\right)$$

where a parsimony (regularisation) parameter ${\lambda}_{comp}$ controls the trade-off between accuracy and simplicity. During the GP evolution, free numerical constants are treated as ephemeral random constants and are inherited/mutated together with the expression tree. To keep the search computationally tractable, we do not perform gradient-based constant fitting for every individual during evolution. Instead, constant refinement is deferred to the model-selection stage (Section "Model Selection"), where only the level-wise winners are optimised using the Limited-memory Broyden-Fletcher-Goldfarb-Shanno (L-BFGS) optimisation algorithm [52], owing to its proven efficacy in parameter estimation and optimisation tasks [53]. Lower values of $\mathcal{F}\left({f}_{\theta }\right)$ indicate better models.

The evolutionary loop applies standard GP [54]: parents are chosen by tournament selection, offspring are produced by subtree crossover with probability ${p}_{c}$ or subtree mutation with probability ${p}_{m}=1-{p}_{c}$, and tree length is capped to control bloat.

All offspring produced through crossover or mutation are again subjected to the same hard-constraint screening before entering the next generation. Throughout the search, individuals are grouped by their structural complexity μ, and fitness competition is performed within each complexity level. This ensures that simpler expressions are never forced to compete directly with far more expressive ones, a mechanism shown to preserve interpretability and prevent unnecessary structural inflation in SR [24, 25, 34]. Once the termination criterion is met (typically a fixed number of generations ${N}_{g}$ or stagnation of the best fitness), the algorithm outputs a level-wise set of candidate models $\mathcal{M}$, where each admissible structural complexity μ retains its best-performing expression

13 $${f}_{\uptheta }^{*\left(\mu \right)}=\mathrm{arg}\underset{{f}_{\theta }\in {\mathcal{F}}_{\mu }}{\mathrm{min}}\mathcal{F}\left({f}_{\theta }\right)$$

where ${\mathcal{F}}_{\mu }$ denotes the subset of feasible expressions (i.e., those satisfying the hard constraints) with structural complexity μ.

After the GP search terminates, the collection

14 $$\mathcal{M}={\left\{{f}_{\uptheta }^{*\left(\mu \right)}\right\} }_{\mu ={\mu}_{\mathrm{m}\mathrm{i}\mathrm{n}}}^{{\mu}_{max}}$$

serves as the candidate model set $\mathcal{M}$ for final selection, where ${\mu}_{\mathrm{m}\mathrm{i}\mathrm{n}}$ denotes the minimum allowed complexity level (default 1), and ${\mu}_{max}$ is the maximum admissible complexity level observed among the retained level-wise winners.

**Fig. 3:**
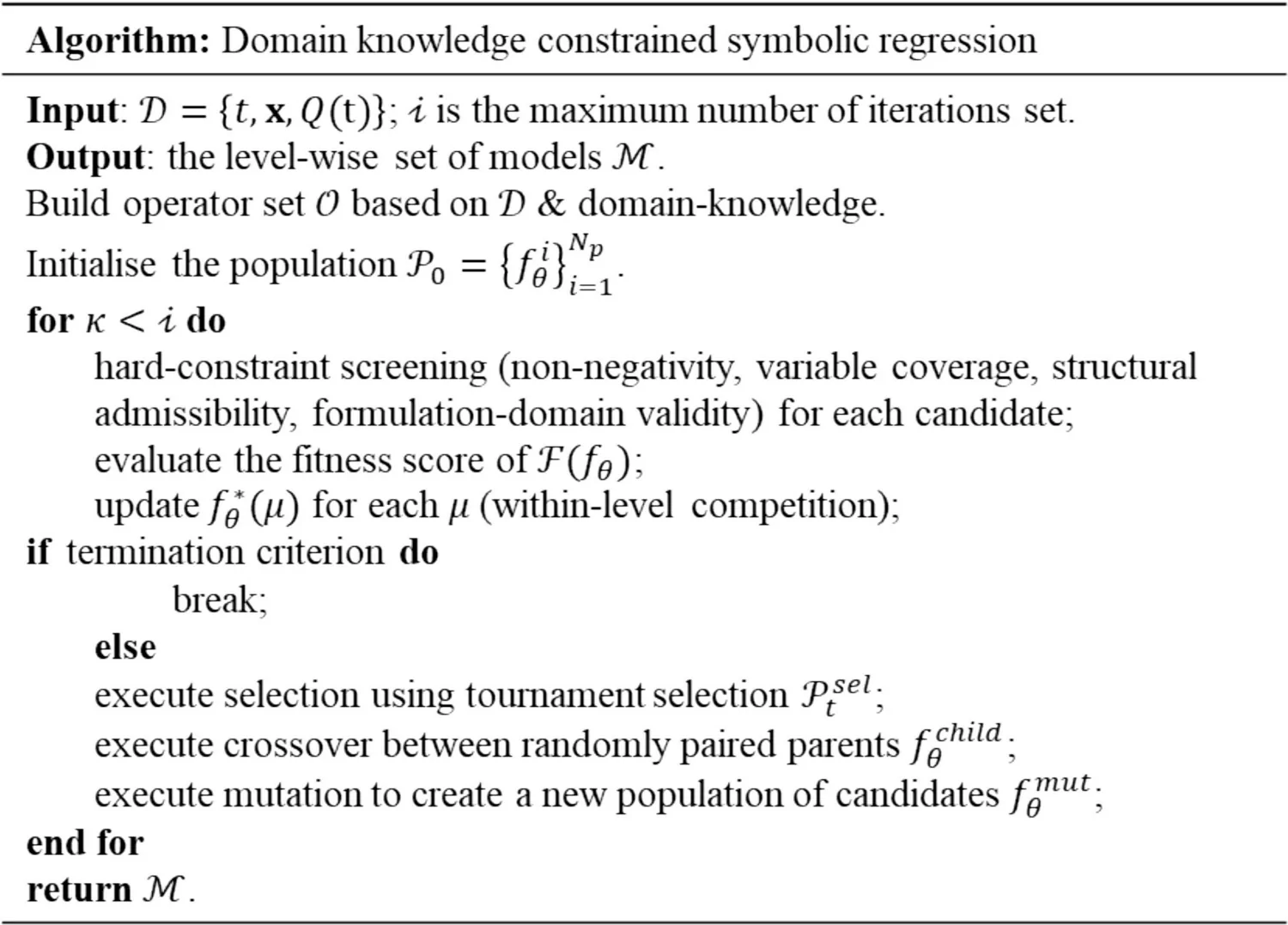
The pseudocode of domain knowledge constrained symbolic regression.

The pseudocode of the evolutionary procedure is provided in Fig. 3.

### Model Selection

Models in the level-wise set $\mathcal{M}$ have already passed feasibility screening, so the objective of this stage is not to ensure validity, but to select the model that best balances goodness-of-fit and parsimony.

For each level-wise best expression ${f}_{\theta }^{*\left(\mu \right)}\in \mathcal{M}$, we first refine its free numerical constants by solving

15 $${\theta}_{\mu }^{*}=\text{arg }\underset{\uptheta }{\mathrm{min}}{\mathcal{L}}_{data}\left({f}_{\theta }^{*\left(\mu \right)}\right)$$

where ${\mathcal{L}}_{data}$ is defined in Eq. (9), and the model predictions are obtained by numerically solving the ODE. The vector of free numerical constants θ is refined by minimising ${\mathcal{L}}_{\mathrm{d}\mathrm{a}\mathrm{t}\mathrm{a}}$, where each objective evaluation requires solving the induced ODE (Eq. (10)). An optimiser, L-BFGS with box constraints on θ, is adopted to improve numerical stability, initialised from the constants obtained at the end of GP evolution (warm start). Gradients are approximated by finite differences. The refinement is applied only to the level-wise winners in $\mathcal{M}$ (rather than to every individual during evolution), keeping the overall computational cost manageable. If the optimiser fails to converge, the original constants were retained.

The AIC [55] is adopted to select among the refined candidates, which is based on the negative log-likelihood [56] under an i.i.d. Gaussian residual assumption:

16 $$\mathrm{A}\mathrm{I}\mathrm{C}\left({f}_{\theta }^{*\left(\mu \right)}\right)={N}_{tot}\mathrm{ln}\left(\frac{{\mathcal{L}}_{data}\left({f}_{\theta }^{*\left(\mu \right)}\right)}{{N}_{tot}}\right)+2{d}_{\mu }$$

where ${N}_{tot}=N\times n$ denotes the total number of observations, and ${d}_{\mu }=\mathrm{d}\mathrm{i}\mathrm{m}({\theta}_{\mu })$ is the total number of estimated parameters (i.e., the number of free constants optimised during the refinement step). Lower AIC values indicate models that achieve a better trade-off between fit quality and parameter parsimony.

The final chosen model is therefore

17 $${f}_{\uptheta }^{final}=\mathrm{arg}\underset{f\in \mathcal{M}}{\mathrm{min}}\mathrm{A}\mathrm{I}\mathrm{C}\left(f\right)$$

This selection strategy evaluates only the best expressions at each complexity level μ and ranks these winners using an information criterion, ensuring that the final model remains interpretable and statistically efficient.

### Formulation Optimisation

At this stage, optimisation is not intended as an exhaustive search for a global optimum, but as a single-round decision-support exercise to examine whether the discovered symbolic model can consistently guide formulations toward high-performing regions of the design space. Once the final dynamic model ${f}_{\theta }^{final}$ has been selected, it is used as a surrogate to optimise the formulation composition within the predefined design space. For a given formulation vector $\mathbf{x}$, the cumulative release profile ${\widetilde{Q}}_{\mathrm{s}\mathrm{r}}\left(t;\mathbf{x}\right)$ is obtained by numerically solving

18 $$d\widetilde{Q}/dt={f}_{\theta }^{final}\left(\widetilde{Q},\mathbf{x}\right), \widetilde{Q}\left(0\right)=0$$

over the experimental time horizon $[0,{t}_{\mathrm{f}}]$, where ${t}_{\mathrm{f}}$ denotes the final sampling time. In this work, the optimisation objective focuses exclusively on the cumulative release at the final time point, ${\widetilde{Q}}_{\mathrm{s}\mathrm{r}}\left({t}_{\mathrm{f}};\mathbf{x}\right)$, rather than the full transient trajectory. This choice is consistent with the experimental endpoint of interest and directly reflects the performance metric typically used to compare formulations.

The resulting single-objective optimisation problem can be written as

19 $$\underset{\mathbf{x}\in \mathcal{X}}{\mathrm{max}}\widetilde{J}\left(\mathbf{x}\right)={\widetilde{Q}}_{\mathrm{s}\mathrm{r}}\left({t}_{\mathrm{f}};\mathbf{x}\right)$$

where $\mathcal{X}$ denotes the feasible formulation design space, defined by lower and upper bounds on each component along with any additional constraints, e.g., concentration limits or practical feasibility conditions.

Since $\widetilde{J}\left(\mathbf{x}\right)$ is obtained by integrating a nonlinear ODE, the objective landscape is generally non-convex, potentially multi-modal, and derivative-free. To address these challenges, the optimisation is performed using the CMA-ES, a robust derivative-free optimiser suitable for non-convex black-box objectives arising from ODE-based model evaluations [32]. CMA-ES maintains a multivariate normal search distribution $\mathcal{N}\left({m}_{t},{\sigma}_{t}^{2}{C}_{t}\right)$ over the decision variables $\mathbf{x}$, where ${m}_{t}$ is the mean, ${\sigma}_{t}$ is the global step size, and ${C}_{t}$ is the covariance matrix at iteration t. At each iteration, a population of candidate formulations $\left\{{\mathbf{x}}^{\left(k\right)}{\}}_{k=1}^{\lambda }\right.$ is sampled from this distribution, evaluated via the surrogate objective $\widetilde{J}\left({\mathbf{x}}^{\left(k\right)}\right)$, and then used to update ${m}_{t}$, ${\sigma}_{t}$, and ${C}_{t}$ based on the ranking of objective values. The algorithm iteratively adapts the search distribution toward high-performing regions of $\mathcal{X}$, with termination triggered by a maximum number of iterations or convergence of the step size [33].

In summary, the combination of the symbolic ODE model ${f}_{\theta }^{final}$ and CMA-ES enables direct optimisation of formulation composition with respect to the predicted cumulative release at the final experimental time, while fully respecting the design-space constraints and the inferred release dynamics.

## Results and Discussion: Model Discovery and Optimisation of Ibuprofen-loaded Poloxamer 407-based Formulations

In this section, to demonstrate the performance and practical utility of the proposed method, we conducted a case study on ibuprofen dermal release using IVRT data from the ibuprofen-loaded poloxamer 407-based formulations. Section "Dynamic Model Discovery and Selection" reports the model derivation, selection, and diagnostic evidence of its suitability for optimisation. Section "Evaluation of Formulation Optimisation" presents the optimisation outcomes, experimental validation, and interpretation of the resulting optimum and model behaviour.

### Dynamic Model Discovery and Selection

The dataset used in this study originates from our previous works [35, 36, 57], which reported IVRT results of ibuprofen-loaded poloxamer 407-based formulations (accessible via GitHub repository referenced in Data availability section). Each formulation consisted of three key optimisable excipient concentrations as summarised in Table 1, poloxamer 407 (${x}_{1}$), ethanol (${x}_{2}$), and propylene glycol (${x}_{3}$). The cumulative amount of released ibuprofen across synthetic membrane was recorded over a time grid $t\in \left\{0.5, 1, 2, 3, 4, 5, 6\right\}$ h, producing the observed response variable ${Q}_{\mathrm{o}\mathrm{b}\mathrm{s}}\left({x}_{1},{x}_{2},{x}_{3},t\right)$, and denotes the cumulative drug amount released per unit membrane area at time t.

Comprising 30 formulations, each subjected to four replicate tests, this dataset yields time-series observations of release curves. Prior to model construction, replicates were averaged at each sampling point to mitigate measurement noise. This dataset therefore provides a structured basis for SR: the formulation variables ${x}_{1},{x}_{2},{x}_{3}$ serve as explanatory inputs, while the cumulative release ${Q}_{\mathrm{o}\mathrm{b}\mathrm{s}}$ provides the time-resolved output from which the dynamic model $d\widetilde{Q}/dt={f}_{\theta }\left(\widetilde{Q},{x}_{1},{x}_{2},{x}_{3}\right)$ is inferred.

### Model Generation and Selection

First, 24 formulations were used as the training set to derive an interpretable and robust dynamic model based on the proposed algorithm and remaining 6 were reserved as the testing dataset. SR was performed exclusively on the training set, while the held-out formulations were later used to assess the generalisation performance of the selected model.

The dynamic model was generated using the DKC-SR framework described in Section "Domain Knowledge Constrained Symbolic Regression". The operator set was kept identical to that defined in Eq. (3), combining basic arithmetic operators with a small number of nonlinear functions to allow sufficient flexibility while preserving interpretability. During evolution, feasibility screening and the complexity-penalised fitness were applied as defined in Sections "Hard Constraints for Feasibility Screening"–"GP-based Model Generation and Evaluation".

For this case study, the main GP hyperparameters were chosen to balance search coverage and computational cost and are summarised in Table 2. The configuration follows the general recommendations for symbolic-regression GP while being adapted to the problem scale of the present IVRT dataset.

**Table 2:** GP configuration for DKC-SR

Parameter	Value
Population size (${N}_{p}$)	4000
Number of generations (${N}_{g}$)	250
Crossover probability $({p}_{c})$	0.6
Mutation probability $({p}_{m})$	0.4
Tournament size (k)	5
Maximum tree length ${(L}_{max})$	25
Maximum initial depth ${(D}_{\mathrm{m}\mathrm{a}\mathrm{x}})$	4
Complexity weight ${(\lambda }_{\mathrm{c}\mathrm{o}\mathrm{m}\mathrm{p}})$	$5\times {10}^{-4}$

In particular, the complexity weight ${\lambda}_{\mathrm{c}\mathrm{o}\mathrm{m}\mathrm{p}}$ was set to $5\times {10}^{-4}$, providing a mild parsimony pressure that discourages unnecessarily complex trees without overwhelming the data-fitting term in Eq. (12).

Under this configuration, the level-wise best models obtained from the GP run are summarised in Table 3. For each structural complexity level μ, the table reports the penalised fitness score, the normalised mean squared error (MSE), and the corresponding AIC value (computed after L-BFGS refinement of the level-wise winners). It is worth noting that no admissible models with complexity μ<15 were retained in the level-wise set $\mathcal{M}$. For these lower-complexity levels, either no expressions satisfied all hard constraints, or the best feasible candidates exhibited larger residual errors and were therefore excluded from consideration. Higher-complexity expressions (e.g., μ>22) were produced during evolution but were rejected during hard-constraint screening and are not included in $\mathcal{M}$. As a result, the retained model set $\mathcal{M}$ corresponds to admissible complexity levels with ${\mu}_{\mathrm{m}\mathrm{i}\mathrm{n}}=15$ to ${\mu}_{\mathrm{m}\mathrm{a}\mathrm{x}}=22$.

**Table 3:** Summary of level-wise best models used in the final selection

No	Fitness Score	Complexity	MSE	AIC	Model
1	0.0351	15	0.0215	−645.02	$\frac{\mathrm{l}\mathrm{n}\left(1+{e}^{ln\left(1+{e}^{\widetilde{Q}}\right)}\right)}{\mathrm{l}\mathrm{n}\left.\left(1+{e}^{{\left.\left(\left.\left({\widetilde{Q}}^{2}- \mathrm{l}\mathrm{n}\left(1+{e}^{{x}_{2}+ {x}_{3}}\right)\right.\right)-{x}_{1}\right.\right)}^{2}}\right.\right)}$
2	0.0344	16	0.0204	−654.27	$\frac{\mathrm{l}\mathrm{n}\left(1+{e}^{ln\left(1+{e}^{\widetilde{Q}}\right)}\right)}{\mathrm{l}\mathrm{n}\left.\left(1+{e}^{{\left.\left(\left({x}_{2}+ {x}_{3}\right)-\left({\widetilde{Q}}^{2}+ {x}_{1}{x}_{2}\right)\right.\right)}^{2}}\right.\right)}$
3	0.0202	17	0.0067	−839.90	$\frac{\mathrm{l}\mathrm{n}\left(1+{e}^{2\widetilde{Q}}\right)}{{\left.\left({x}_{2}-\left.\left({x}_{1}/{x}_{3}-{\widetilde{Q}}^{2}\right.\right)\right.\right)}^{2}+2.355}$
4	0.0202	18	0.0249	−618.12	$\frac{\mathrm{l}\mathrm{n}\left(1+{e}^{2\widetilde{Q}}\right)}{\mathrm{l}\mathrm{n}\left(1+{e}^{-0.827}\right)+\mathrm{l}\mathrm{n}\left.\left(1+{e}^{{\left.\left({x}_{2}- \left.\left({x}_{1}/{x}_{3}- {\widetilde{Q}}^{2}\right.\right)\right.\right)}^{2}}\right.\right)}$
5	0.0207	19	0.0069	−834.67	$\frac{\mathrm{l}\mathrm{n}\left(1+{e}^{2\widetilde{Q}}\right)}{\widetilde{Q}+{\left.\left({x}_{2}-\left.\left({x}_{1}/{x}_{3}- {\widetilde{Q}}^{2}\right.\right)\right.\right)}^{2}+\mathrm{l}\mathrm{n}\left(1+{e}^{0.227}\right)}$
6	0.0347	20	0.0186	−669.19	$\frac{\mathrm{l}\mathrm{n}\left(1+{e}^{2\widetilde{Q}}\right)}{{\widetilde{Q}}^{2}+\mathrm{l}\mathrm{n}\left.\left(1+{e}^{{\left.\left({x}_{2}-\left.\left({x}_{1}/{x}_{3}-\left({\widetilde{Q}}^{2}+ \widetilde{Q}\right)\right.\right)\right.\right)}^{2}}\right.\right)}$
7	0.0360	21	0.0202	−655.52	$\frac{\mathrm{l}\mathrm{n}\left(1+{e}^{\widetilde{Q}}\right)}{\mathrm{l}\mathrm{n}\left.\left(1+{e}^{\left.\left(\mathrm{l}\mathrm{n}\left(1+{e}^{{x}_{3}}\right)-\mathrm{l}\mathrm{n}\left(1+{e}^{{x}_{1}+ {x}_{3}}\right)\right.\right)-{\left.\left({x}_{2}-\left({\widetilde{Q}}^{2}+ {x}_{1}{x}_{2}\right)\right.\right)}^{2}}\right.\right)}$
8	0.0346	22	0.0209	−649.47	$\frac{\mathrm{l}\mathrm{n}\left(1+{e}^{\mathrm{l}\mathrm{n}\left(1+{e}^{\widetilde{Q}}\right)}\right)}{\mathrm{l}\mathrm{n}\left.\left(1+{e}^{{\left.\left({x}_{3}{x}_{2}-\left.\left(\left({x}_{2}+ {x}_{3}\right)- \left({\widetilde{Q}}^{2}-0.129\right)+{x}_{1}{x}_{3}\right.\right)\right.\right)}^{2}}\right.\right)}$

As shown in Table 3, both the fitness score and the MSE improve markedly when the complexity increases from $\mu =15 \mathrm{a}\mathrm{n}\mathrm{d} 16$ to μ=17, indicating that very simple structures are insufficient to capture the observed release dynamics. Beyond μ=17, further increases in complexity lead to only marginal changes in MSE, while incurring additional structural cost. Consequently, the AIC identifies model No. 3 with μ=17 as the most statistically efficient, reflecting a favourable trade-off between fit quality and parsimony. This model is therefore selected as the final rate expression ${f}_{\theta }^{final}$ and subsequently employed for formulation optimisation.

#### Surrogate Diagnostics and Optimisation Setup

**Fig. 4:**
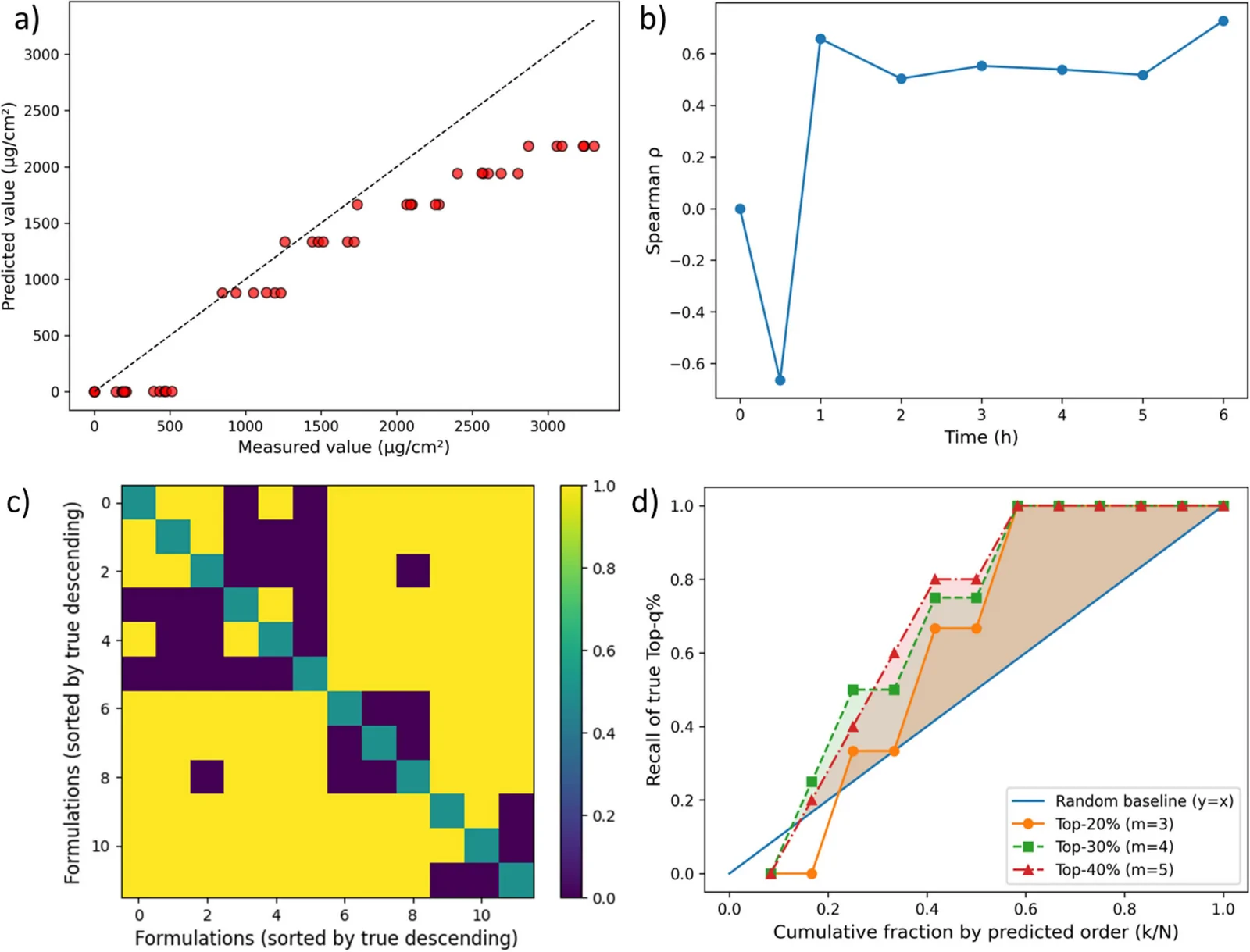
Diagnostic evaluation of the final dynamic model and its suitability for formulation optimisation. (**a**) The performance of discovered model on the test set. (**b**) Spearman rank correlation between predicted and observed cumulative release as a function of time. (**c**) Pairwise ordering agreement for ${Q}_{obs}(6 h)$ across 12 formulations. (**d**) Gain curves showing the recall of the true top-$q \mathrm{\%}$ formulations as a function of the fraction of formulations inspected in the predicted order; the diagonal line indicates the random baseline.

After selecting the final model, we examined its held-out pointwise accuracy primarily to characterise its limitations before using it as a ranking-oriented dynamic surrogate for optimisation. Figure 4 summarises several diagnostic assessments of the model on cumulative release. Figure 4a shows the performance of predicted *versus* measured cumulative release for the six test formulations. The model achieves ${\mathrm{R}}^{2}=0.80$ with a root mean squared error (RMSE) of 461.26 $\upmu \mathrm{g}/{\mathrm{cm}}^{2}$, indicating that the model captures broad between-formulation variation in release and is sufficiently informative for downstream ranking-based optimisation, even though exact quantitative agreement remains limited.

Because the subsequent optimisation relies on the relative ranking of formulations rather than on perfectly accurate point predictions, additional rank-based diagnostics were computed. The held-out test set, however, contains only six formulations, which is too small to yield stable rank statistics; restricted to the test set alone, such statistics are unstable and cannot be reliably estimated. We therefore computed the rank diagnostics on a combined set of the six test and six representative training formulations (12 in total). Incorporating training data stabilises the estimates but introduces some optimism, so these diagnostics are reported as supportive rather than confirmatory evidence (see Section "Evaluation of Formulation Optimisation", where the optimisation outcome is assessed experimentally). Figure 4b reports the Spearman rank correlation [58] between predicted and observed cumulative release as a function of time. The correlation is weak at the earliest sampling points but becomes clearly positive from 1 h onwards and is highest at 6 h, indicating that the model provides a positive ranking of formulations at the final time point (ρ=0.73), which is the optimisation target.

To further examine pairwise ordering, Fig. 4c presents a pairwise agreement heatmap [59] for ${Q}_{obs}(6 h)$. Among the 66 possible formulation pairs, 50 are correctly ordered by the surrogate, corresponding to a pairwise concordance of 0.76 ($p=1.7\times {10}^{-5}$). The high proportion of correctly ordered pairs, particularly among higher-performing formulations, supports the use of the surrogate for prioritising candidates on this stabilised set, even where point predictions are imperfect.

Finally, Fig. 4d shows gain curves that quantify the enrichment of high-release formulations when they are selected according to the surrogate predictions. The curves display the recall of the true top-$q \mathrm{\%}$ formulations (q=20,30,40) as a function of the fraction of formulations inspected in the predicted order. In all cases, the gain curves lie above the random baseline, demonstrating that the surrogate substantially enriches the discovery of high-release formulations. Based on these enrichment profiles and practical laboratory constraints, we decided to select the top 5 optimised solutions by the surrogate as a compromise between experimental cost and expected improvement.

The final optimisation step then employed CMA-ES with ${f}_{\theta }^{final}$ as the surrogate objective, following the formulation described in Section "Formulation Optimisation". CMA-ES was initialised within the bounded design space $\mathcal{X}$ defined by the concentration limits of poloxamer 407, ethanol and PG in Table 1, and iteratively adapted its search distribution to explore high-performing regions of this space. CMA-ES was run with a population size 50, an initial step size 0.2, and a maximum of 100 iterations, using common default adaptation parameters as recommended in [32].

### Evaluation of Formulation Optimisation

#### Optimised Formulation and Experimental Validation

**Fig. 5:**
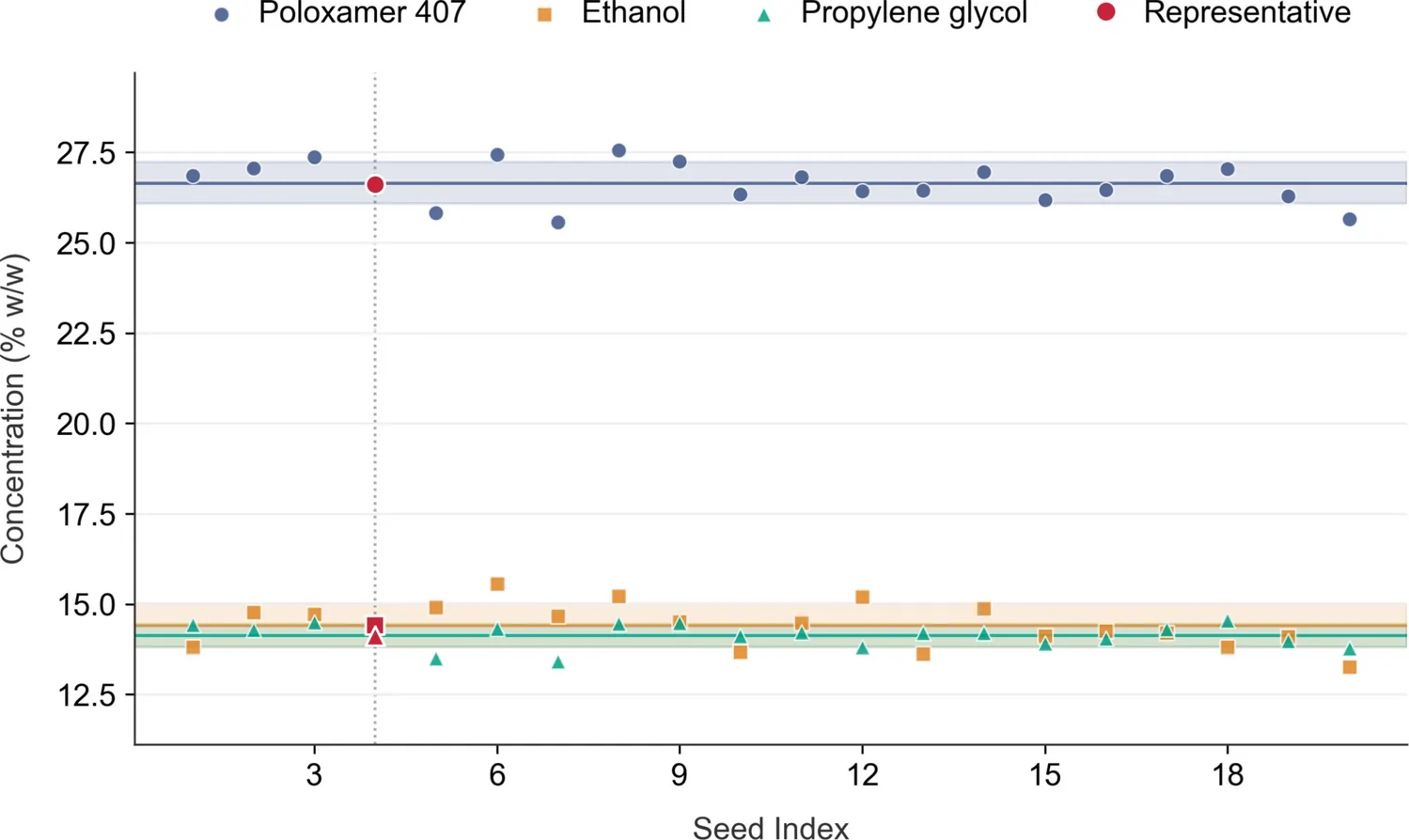
Best solution concentrations across 20 CMA-ES restarts with different random seeds.

After convergence, CMA-ES concentrated its final population in a narrow neighbourhood in the design space, indicating a locally flat predicted optimum on the surrogate objective. To examine the robustness to stochasticity, we repeated CMA-ES with 20 different random seeds on the trained surrogate. All runs converged and returned near-identical predicted optima (mean is $Q(6 \mathrm{h})\approx 2425.75 \text{ } \upmu \mathrm{g}/{\mathrm{cm}}^{2}$, standard deviation is $7.77\times {10}^{-8}$). Meanwhile, the corresponding concentrations clustered within a narrow neighbourhood in Fig. 5, indicating a locally flat optimum (plateau/ridge) on the surrogate objective rather than a chance outcome from a single run.

Given this near-degeneracy of the predicted optima, validating multiple restart-specific solutions would provide limited additional information at the formulation level. We therefore report a representative optimum, selected as the restart-best closest to the multi-seed mean in design space in Fig. 5, and use it as the reference solution for subsequent analysis and experimental evaluation (conducted in four replicates). Table 4 summarises the multi-seed statistics and representative concentration together with its predicted objective and the corresponding measured outcome. Although Section "Surrogate Diagnostics and Optimisation Setup" motivates selecting a small set of top-ranked candidates when distinct optima exist, the present case exhibits a plateau on the surrogate objective, under which the top-ranked solutions become near-degenerate for each restart; hence we adopt a representative-solution reporting strategy here.

**Table 4:** Multi-seed restart summary and representative solution

Item	Poloxamer 407 (%w/w)	Ethanol (%w/w)	Propylene glycol (%w/w)	Prediction ($\upmu \mathrm{g}/{\mathrm{cm}}^{2}$)	Measured ($\upmu \mathrm{g}/{\mathrm{cm}}^{2}$)
Multi-seed mean ± SD	26.65±0.58	14.41±0.60	14.13±0.32	$2425.75 \pm 7.77\times {10}^{-8}$	-
Representative optimum	26.62	14.42	14.11	2425.75	3445.89±295.46

**Fig. 6:**
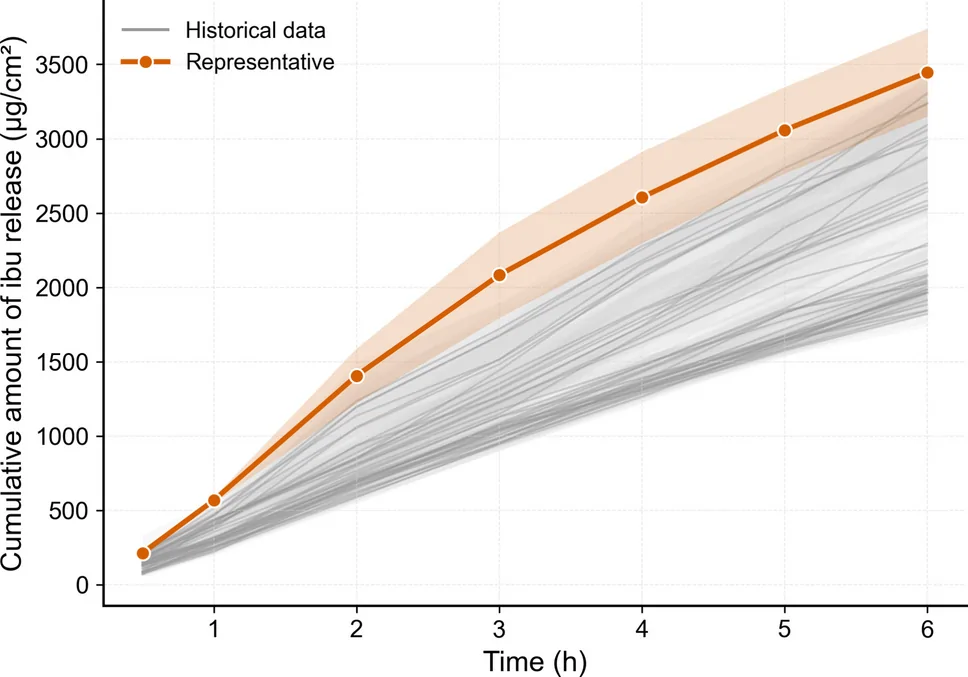
IVRT release profiles of ibuprofen for all historical formulations (grey curves) and the representative optimum (orange curve).

Compared with the historical best formulation from the original dataset (3305.14 ± 116.78 $\upmu \mathrm{g}/{\mathrm{cm}}^{2}$), the representative formulation selected from the CMA-ES plateau achieved a higher cumulative release at 6 h (3445.89 ± 295.46 $\upmu \mathrm{g}/{\mathrm{cm}}^{2}$), corresponding to an improvement of approximately 4%. This behaviour is consistent across the entire 0.5–6 h time window as shown in Fig. 6, the representative release profiles track the upper envelope of the historical IVRT curves and remain among the highest-releasing profiles throughout the experiment. The measured value is substantially higher than the surrogate prediction reported in Table 4, indicating that the current model is better suited to ranking and identifying high-performing regions than to exact quantitative calibration of the final release magnitude. Despite the imperfect absolute prediction accuracy of the model, the domain knowledge constrained surrogate provides sufficient decision utility to steer the optimiser toward a high-performing region of the formulation space and to prioritise experimentally actionable candidates.

Because the optimisation was performed in a single round using a fixed surrogate model, repeated optimiser restarts are not expected to produce meaningfully distinct formulations; instead, they converge to a near-degenerate optimum neighbourhood. Further improvement would therefore require incorporating newly generated experimental data and retraining the symbolic regression model, enabling subsequent optimisation rounds to exploit the updated surrogate.

Beyond quantitative performance, the selected model also remains interpretable in structural and functional terms. The softplus term in the numerator, $softplus\left(2\widetilde{Q}\right)=\mathrm{l}\mathrm{o}\mathrm{g}(1+\mathrm{e}\mathrm{x}\mathrm{p}(2\widetilde{Q}))$, has a clear modelling role: it enforces a smooth non-negative fitted release rate, and approximates an initially increasing rate which gradually transitions to an approximately linear growth (with bounded slope) as $\widetilde{Q}$ grows, consistent with a diminishing thermodynamic driving force as the donor phase is depleted. The denominator,

20 $${\left.\left({x}_{2}- \left.\left({x}_{1}/{x}_{3}- {\widetilde{Q}}^{2}\right.\right)\right.\right)}^{2}+\kappa$$

couples the cumulative release $\widetilde{Q}$ with the relative levels of ethanol, propylene glycol and poloxamer. Here $\widetilde{Q}$ and ${x}_{1},{x}_{2},{x}_{3}$ are the normalised release and the [0,1]-scaled concentrations defined in Section "Problem Setting for ODE-based Dynamic Model Discovery"; κ=2.355 (95% bootstrap interval 2.212–2.942; Supplementary S3) is the only free fitted constant, while the factor 2 in softplus($2\widetilde{Q}$) is structural ($\widetilde{Q}+\widetilde{Q}$) and was not fitted. This structure indicates that the fitted release rate depends jointly on the system state and formulation composition. In this sense, the expression provides a compact phenomenological structure that is qualitatively consistent with empirical expectations for poloxamer-based cosolvent systems while remaining stable and interpretable for optimisation.

#### Model Comparison, Sensitivity, and Robustness Analysis

To evaluate the selected symbolic surrogate more comprehensively, we examined its performance relative to same-split baseline models, its stability under fixed-structure bootstrap refitting, the sensitivity of the SR stage to GP hyperparameters, and the local behaviour of the dynamic surrogate around the optimised formulation.

Baseline models were trained and evaluated using the same formulation-level validation split as the selected DKC-SR model. For curve-level prediction, the selected DKC-SR equation achieved a test curve RMSE of 461.26 and ${\mathrm{R}}^{2}=0.80$, which was lower than the curve-proxy baselines, including PLS regression, ridge regression, random forest regression, polynomial response surface regression (RSM), and Gaussian process regression. The best vanilla ODE-SR seed achieved a test curve RMSE of 549.95. The selected DKC-SR equation also achieved the lowest area under the curve (AUC) RMSE among the compared models, indicating better integrated release-profile fidelity over the full observation window. These results indicate that the selected DKC-SR equation provides competitive dynamic curve prediction under the same held-out split.

The endpoint results showed a different pattern. PLS regression achieved a lower $Q(6 \mathrm{h})$ endpoint RMSE than the selected DKC-SR equation on the six held-out test formulations. This indicates that conventional regression models remain useful endpoint comparators. However, unlike the baselines, DKC-SR provides an explicit symbolic ODE release-rate expression that can be replayed dynamically and coupled with formulation optimisation. The vanilla ODE-SR baseline also provided a relevant symbolic comparison, but the tested vanilla seeds collapsed to formulation-independent expressions, limiting their utility as formulation optimisation surrogates (Table 5).

**Table 5:** Same-split baseline comparison on the six held-out test formulations

Model	Model type	Test curve RMSE	Test curve (${\mathrm{R}}^{2}$)	$Q(6 \mathrm{h})$ RMSE	AUC RMSE	Dynamic ODE surrogate	Optimisation usability
DKC-SR selected equation	Constrained symbolic ODE	461.26	0.80	959.38	1764.26	Yes	Yes
PLS regression	Regression	507.96	0.75	803.03	2249.05	No	Endpoint/curve proxy
Ridge regression		510.84	0.75	811.34	2274.36		
Random forest regression		529.56	0.73	936.83	2385.66		
Polynomial RSM		538.99	0.72	847.47	2568.87		
Gaussian process		590.92	0.67	979.99	2795.76		
Best vanilla ODE-SR seed	Generic symbolic ODE	549.95	0.71	914.48	2665.01	Yes	Limited; formulation-independent

Vanilla ODE-SR used generic arithmetic primitives and did not use the DKC-SR constraint strategy. Full metric tables and seed-level results are provided in the Supplementary Information S1, S2 & S3

The stability of the selected symbolic structure was further examined using fixed-structure bootstrap refitting. The final DKC-SR expression was kept unchanged, with ($\mathrm{s}\mathrm{o}\mathrm{f}\mathrm{t}\mathrm{p}\mathrm{l}\mathrm{u}\mathrm{s}(2\widetilde{Q})$) treated as part of the discovered symbolic structure and only the denominator offset (κ) refitted. Across 500 bootstrap resamples of the 24 training formulations, all refits were successful. The refitted (κ) values remained within a bounded range, with ($\kappa =2.564\pm 0.199$) and a 95% interval of 2.212–2.942. The selected final-equation value (κ=2.355) lay within this interval. The bootstrap test curve RMSE was (499.99±37.03), indicating moderate prediction variability under resampling but no failed refitting or numerical instability. These results suggest that the selected symbolic structure can be refitted consistently under small-data resampling, although the remaining uncertainty reflects the limited number of available formulations. Full bootstrap refitting results are provided in Supplementary Material S3.

**Fig. 7:**
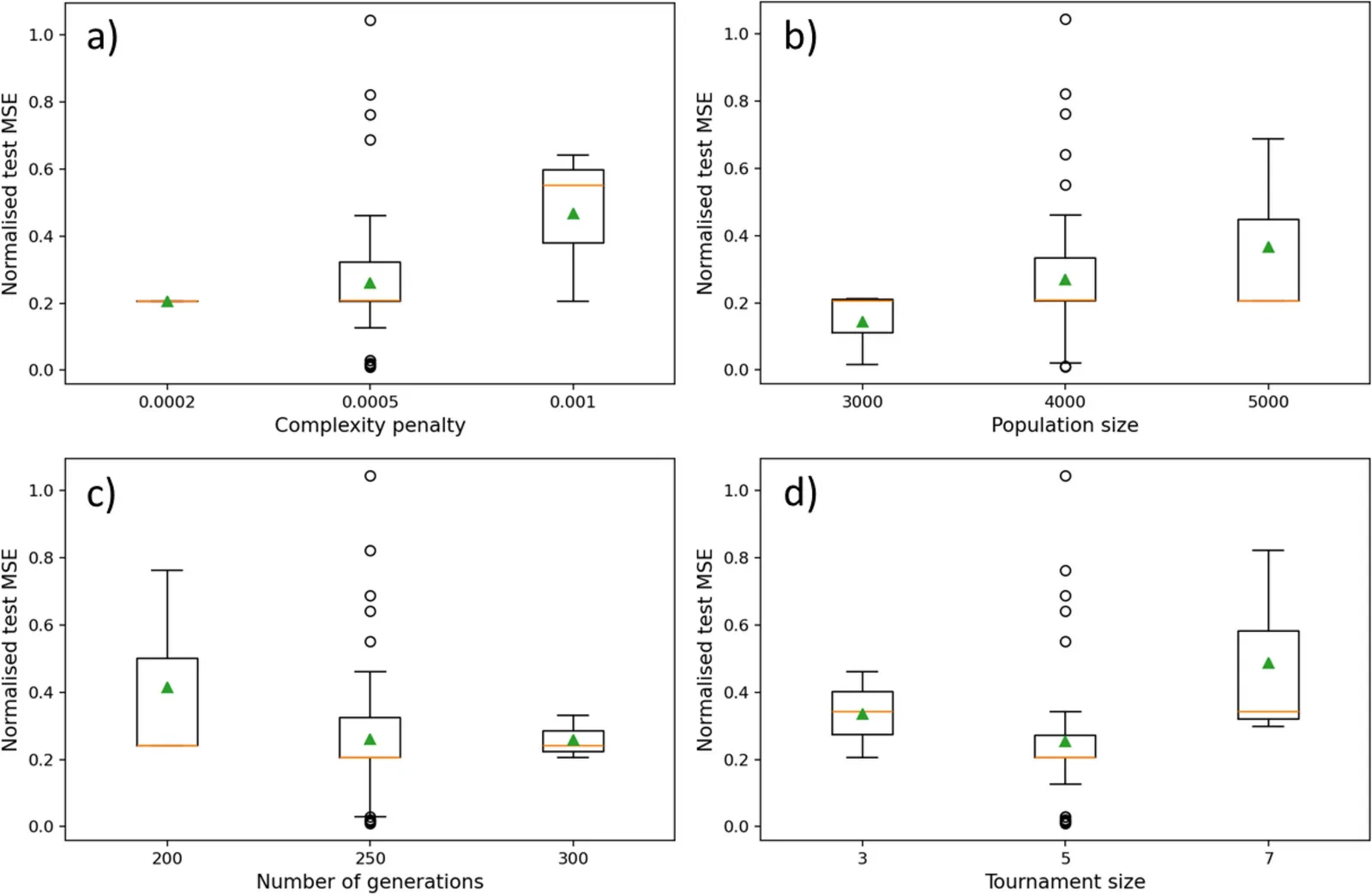
Effect of key GP hyperparameters on SR performance. Boxplots of normalised test MSE for different settings of (**a**) complexity penalty, (**b**) population size, (**c**) number of generations, and (**d**) tournament size, with all other hyperparameters fixed at their baseline values. Each box summarises three independent runs with different random seeds.

We next performed a one-at-a-time hyperparameter study around the baseline configuration in Table 2. Population size, number of generations, crossover probability, tournament size, maximum tree length, maximum initial depth, and the complexity penalty weight ${\lambda}_{comp}$ were each varied across three candidate values, while all other settings were held fixed. Each configuration was repeated with three independent random seeds, and normalised test MSE of the best model was recorded. Figure 7 summarises the four most influential hyperparameters. Overall, performance was stable under moderate changes in GP settings, suggesting the framework does not rely on fragile tuning. The clearest effect was from ${\lambda}_{comp}$: small-to-moderate values of ($2\times {10}^{-4}\sim 5\times {10}^{-4})$ yielded consistently low errors, whereas an excessively strong penalty (${10}^{-3}$) degraded performance, consistent with over-regularisation. Changes in population size and generations (Fig. 7b & c) primarily affected variability rather than the central tendency of the test error: increasing the population from 3000 to 5000 or the number of generations from 200 to 300 produced only modest improvements relative to the run-to-run variability induced by the random seed, but at a higher computational cost. Tournament size (Fig. 7d) showed a more distinct optimum, with an intermediate value (5) outperforming both weaker (3) and stronger (7) selection pressure, aligning with common GP practice. The remaining hyperparameters exhibited only minor effects compared with random-seed variability, indicating that the baseline in Table 2 lies in a reasonably robust operating region.

**Fig. 8:**
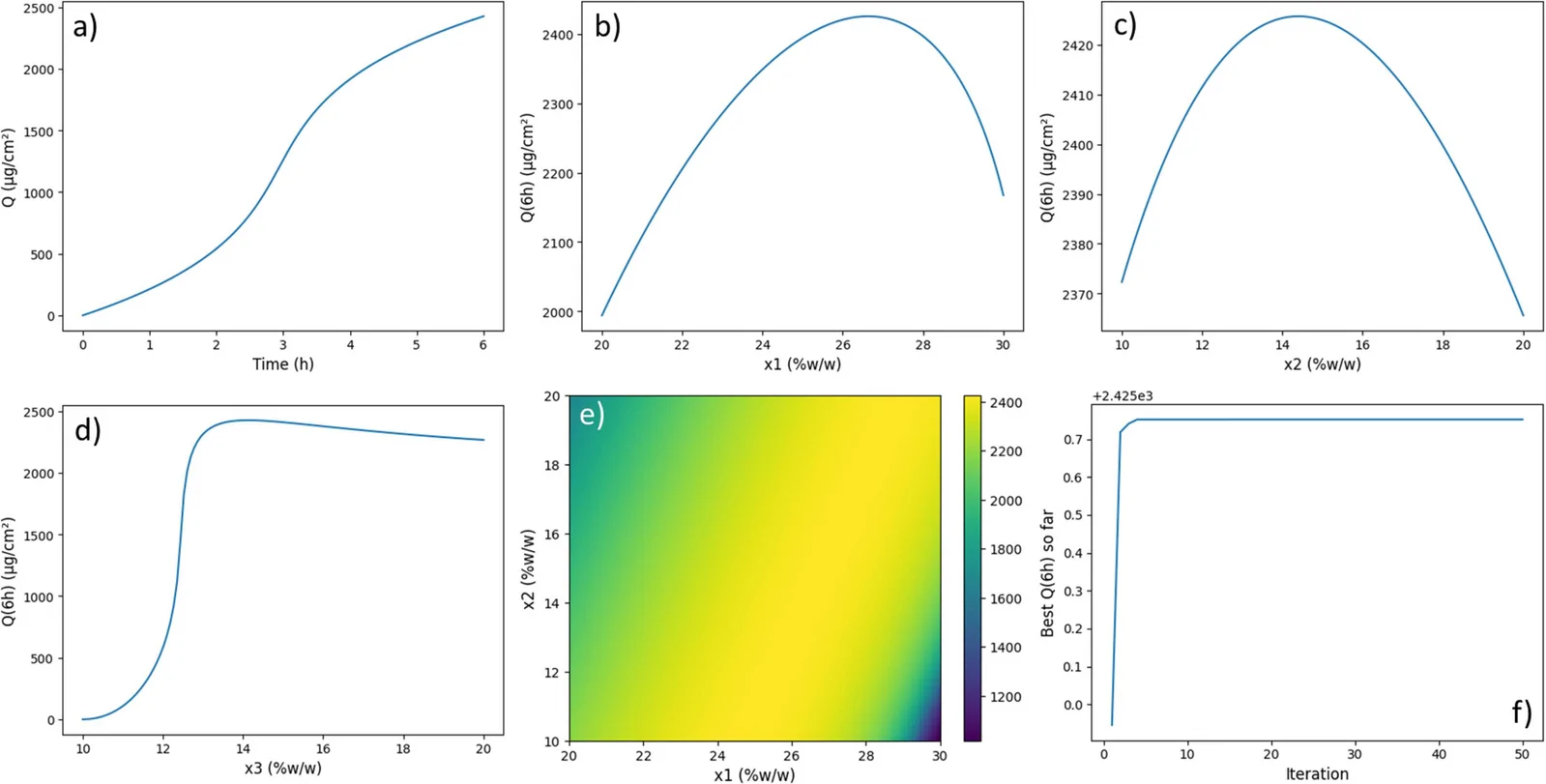
Local response and optimisation diagnostics around the CMA-ES–optimised formulation. (**a**) Predicted cumulative release profile Q for the representative optimum. (**b**–**d**) One-dimensional sensitivity of $Q(6 \mathrm{h})$ with respect to poloxamer 407 (${x}_{1}$), ethanol (${x}_{2}$) and propylene glycol (${x}_{3}$), respectively, evaluated around the optimum. (**e**) Two-dimensional response surface of $Q(6 \mathrm{h})$ as a function of ${x}_{1}$ and ${x}_{2}$ at fixed ${x}_{3}$.(**f**) Convergence of CMA-ES in terms of the best objective value found over iterations.

In addition to GP robustness, we analysed the local behaviour of the dynamic surrogate around the best CMA-ES solution. All analyses in this part are based on the predicted cumulative release at 6 h, $Q(6 \mathrm{h})$. Figure 8a shows the predicted profile Q for the optimised formulation. The curve is smooth and strictly increasing, with an initially accelerating release that transitions to a slower, almost linear increase after approximately 3 h. This trajectory is consistent with diffusion-controlled release under a progressively diminishing driving force and supports that the model yields smooth and physically interpretable dynamics at the optimum.

Figure 8b–d depict one-dimensional sensitivity curves obtained by varying each formulation variable while keeping the other two fixed at their optimised values. The response to poloxamer 407 and ethanol are smooth and unimodal with relatively broad maxima, indicating limited sensitivity to moderate perturbations around the chosen levels. In contrast, the response to propylene glycol increases steeply up to the optimum and then plateaus, suggesting a threshold requirement for high release with diminishing returns beyond that point. The two-dimensional surface in Fig. 8e (poloxamer 407 *vs* ethanol at fixed propylene glycol) reveals an extended ridge of high $Q(6 \mathrm{h})$ rather than a sharp isolated peak, confirming a reasonably wide basin of high-performing formulations and practical tolerance to small compositional deviations. Consistent with this landscape, CMA-ES rapidly reaches a high-performing region within a few iterations and then stabilises (Fig. 8f), indicating efficient convergence under the chosen settings.

Overall, the same-split baseline comparison, fixed-structure bootstrap refitting, GP hyperparameter sweeps, and local response diagnostics support that the proposed framework provides a compact and formulation-dependent dynamic surrogate for optimisation.

## Conclusion

This work presents a domain knowledge constrained symbolic regression framework that discovers compact, interpretable ODE-based dynamic models of dermal drug release and uses them as surrogates to support formulation optimisation. Domain knowledge is embedded into model discovery through a numerically robust operator set and explicit feasibility constraints, ensuring candidate expressions remain physically plausible and numerically stable when integrated as ODEs across the experimental domain. In an ibuprofen case study, the framework generated a level-wise set of candidates and selected a parsimonious final expression via an information-criterion trade-off. While the surrogate is not intended to be a perfect point predictor, its ranking, supported on a stabilised set and ultimately confirmed by experiment, was sufficient to prioritise high-performing formulations; combined with CMA-ES, it guided the search towards a narrow high-performing region. Experimental evaluation at a representative optimum from this region confirmed performance near the upper envelope of historical release profiles and above the best historical formulation, demonstrating that SR-discovered dynamics can translate into experimentally actionable formulation guidance.

Two methodological insights emerge. First, for formulation screening under limited data, the most valuable property of a dynamic surrogate may be trend fidelity (i.e., correct prioritisation) rather than minimal absolute error, making rank-based diagnostics a useful complement to conventional regression metrics. Second, the selected symbolic structure showed refit stability under fixed-structure bootstrap refitting, with the final denominator offset remaining within the bootstrap interval. Together with the observed robustness of the GP stage and the optimisation stage to moderate hyperparameter variations, this indicates that the workflow can be deployed without delicate manual tuning, while still requiring cautious interpretation under small-data conditions.

A key limitation in this case study is that the surrogate underestimates the absolute magnitude of the best-performing formulations, while still providing useful relative ordering on the stabilised diagnostic set. In addition, the SR model was fitted to mean release trajectories rather than replicate-level data, so experimental uncertainty was not explicitly incorporated into the optimisation objective. The limited dataset also means that structural uniqueness and parameter calibration cannot be fully established, even though fixed-structure bootstrap refitting supported the stability of the selected equation under resampling. In addition, the held-out test set comprises only six formulations; rank-based diagnostics restricted to this set are statistically unstable, so the rank statistics in Section "Surrogate Diagnostics and Optimisation Setup" rely on a combined test-plus-training set and should be read as optimistic, supportive evidence rather than an unbiased estimate of held-out ranking. The optimisation outcome is validated experimentally rather than by the held-out rank statistic alone. Future work will therefore focus on calibration strategies, such as a global scaling layer or uncertainty-aware correction, larger formulation datasets, and additional experimental rounds to further assess structural reproducibility and improve absolute calibration. Extending the framework to longer observation windows and to *in vitro *permeation test settings will also enable late-time constraints (e.g., saturation or plateau behaviour) to be reintroduced and evaluated. Overall, the proposed approach offers a reproducible route from limited IVRT time-series data to interpretable dynamic models and experimentally validated formulation improvements, with clear pathways for further accuracy gains.

## Supplementary Information

Below is the link to the electronic supplementary material.

ESM 1 (DOCX 27.8 KB)

## Data Availability

The proposed algorithm and its corresponding dataset are available in the GitHub repository: https://github.com/yurbro/Yu-Zhang-PhD/tree/main/DKC-SR.
