# Supplementary material for: Domain Knowledge Constrained Symbolic Regression for Optimising Dermal Drug Formulations
Source: Pharm Res. 2026 Aug 3;43(9):3393–410. doi: 10.1007/s11095-026-04170-9 (PMC13642449; doi:10.1007/s11095-026-04170-9)
Supplement: Supplementary file 1 [file 11095_2026_4170_MOESM1_ESM.docx]

**Domain knowledge constrained symbolic regression for optimising dermal drug formulations**

Yu Zhang^a^, Xilu Wang^b^, Dimitrios Tsaoulidis^a^, Tao Chen^a*^

^a^*School of Chemistry and Chemical Engineering, University of Surrey, Guildford GU2 7XH, UK*

*^b^School of Computer Science and Electronic Engineering, University of Surrey, Guildford GU2 7XH, UK*

*Corresponding author: Tao Chen, School of Chemistry and Chemical Engineering, University of Surrey, Guildford GU2 7XH, UK. E-mail: t.chen@surrey.ac.uk

**S1. Same-split baseline comparison**

To provide same-dataset baseline comparisons, conventional regression models were trained and evaluated using the same formulation-level validation split as the selected DKC-SR model. The baselines included ridge regression, partial least squares (PLS) regression, polynomial response surface regression (RSM), random forest (RF) regression, and Gaussian process regression. These models were included as endpoint and curve-proxy comparators because they predict release responses directly from formulation variables but do not provide an explicit ODE release-rate expression.

All baseline models were evaluated on the same six held-out test formulations. Primary curve-level metrics were calculated after excluding (t=0). Endpoint metrics were calculated separately at the final release time ($Q_{6}$) and for the area under the release curve (AUC).

Table S1. Endpoint-level comparison on the six held-out test formulations.

| Model | $\mathrm{RMS}E_{Q_{6}}$ | $\mathrm{MA}E_{Q_{6}}$ | $R_{Q_{6}}^{2}$ | $\mathrm{RMS}E_{AUC}$ | $\mathrm{MA}E_{AUC}$ | ${R^{2}}_{AUC}$ |
| --- | --- | --- | --- | --- | --- | --- |
| PLS | 803.03 | 782.18 | -29.49 | 2249.05 | 2127.63 | -11.33 |
| Ridge | 811.34 | 791.04 | -30.12 | 2274.36 | 2155.40 | -11.60 |
| Polynomial RSM | 847.47 | 823.96 | -32.96 | 2385.66 | 2255.96 | -12.87 |
| RF | 936.83 | 918.83 | -40.50 | 2568.87 | 2449.60 | -15.06 |
| DKC-SR | 959.38 | 948.21 | -42.52 | 1764.26 | 1642.83 | -6.58 |
| Gaussian process regression | 979.99 | 967.32 | -44.41 | 2795.76 | 2693.32 | -18.05 |

Table S2. Curve-level comparison on the six held-out test formulations.

| Model | Test curve RMSE | Test curve MAE | Test curve (R^2^) | Number of points |
| --- | --- | --- | --- | --- |
| DKC-SR | 461.26 | 325.58 | 0.80 | 42 |
| PLS | 507.96 | 398.96 | 0.75 | 42 |
| Ridge | 510.84 | 401.28 | 0.75 | 42 |
| RF | 529.60 | 420.64 | 0.73 | 42 |
| Polynomial RSM | 538.99 | 428.09 | 0.72 | 42 |
| Gaussian process regression | 590.92 | 470.32 | 0.67 | 42 |

DKC-SR also achieved the lowest AUC RMSE among all comparators, consistent with a more faithful representation of the cumulative release trajectory, whereas PLS achieved the lowest Q(6 h) endpoint RMSE. This split, with DKC-SR favoured on trajectory- and integral-level error and the regression baselines favoured on the single endpoint, supports reporting curve-level and endpoint-level metrics separately, and indicates that DKC-SR is best interpreted as an optimisation-oriented dynamic surrogate rather than a universally lowest-error endpoint predictor.

**S2. Vanilla ODE-SR baseline**

A vanilla ODE-SR baseline was implemented to compare the proposed DKC-SR framework with conventional symbolic ODE discovery under the same formulation-level validation split. The vanilla baseline used the same 24-training/6-test split, and the same ODE replay framework. However, the domain-knowledge constraint strategy used by DKC-SR was removed. The primitive set contained generic arithmetic operations, including addition, subtraction, multiplication, protected division, and squared terms. The softplus primitive was not included.

Five independent vanilla ODE-SR seeds were evaluated. Each successful candidate was replayed on the same six held-out test formulations, and primary curve metrics were calculated after excluding (t=0).

Table S3. Performance of the vanilla ODE-SR baseline.

| Seed | Test curve RMSE | Test curve MAE | Test curve (R^2^) | Q6 RMSE | AUC RMSE | Tree size | Tree depth |
| --- | --- | --- | --- | --- | --- | --- | --- |
| 0 | 549.95 | 439.42 | 0.71 | 914.48 | 2665.01 | 3 | 1 |
| 3 | 570.40 | 457.05 | 0.69 | 949.03 | 2766.99 | 3 | 1 |
| 2 | 576.53 | 462.35 | 0.68 | 959.42 | 2797.69 | 3 | 1 |
| 4 | 578.65 | 464.15 | 0.68 | 962.96 | 2808.14 | 3 | 1 |
| 1 | 588.76 | 472.91 | 0.67 | 980.01 | 2858.54 | 2 | 1 |

Across the tested seeds, the vanilla ODE-SR candidates collapsed to formulation-independent expressions (tree size 2–3) under this small-data setting. Such models cannot serve as formulation-optimisation surrogates regardless of their apparent fit: because the rate law does not depend on the excipient composition, optimisation over ($x_{1}, x_{2}, x_{3}$) is ill-defined, even where the endpoint error is comparable to a composition-dependent model. On the curve-level metric, the best vanilla seed (RMSE=549.95) was also worse than the selected DKC-SR equation (461.26). These results support the role of DKC-SR as a constrained symbolic ODE discovery framework designed to produce compact, formulation-dependent, and dynamically evaluable surrogate equations.

**S3. Fixed-structure bootstrap refitting of the selected symbolic equation**

To further assess the robustness of the selected symbolic structure under the limited-data setting, a fixed-structure bootstrap refitting analysis was performed. The selected DKC-SR expression was kept unchanged throughout this analysis. In particular, the numerator term ($\mathrm{softplus}(2\tilde{Q})$) was treated as part of the discovered symbolic structure, and the coefficient 2 was not refitted as an additional free parameter. Only the denominator offset ($\kappa$) was refitted.

For each bootstrap replicate, the training formulations were sampled with replacement. The denominator offset ($\kappa$) was then refitted by minimising the curve-level error, and the refitted equation was evaluated on the fixed six-formulation held-out test set. This procedure was repeated for 500 bootstrap resamples. Primary curve metrics were calculated after excluding (t=0). No new symbolic-structure search was performed during this analysis.

Across 500 bootstrap resamples, all refits were successful. The refitted ($\kappa$) values had a mean of 2.564 and a standard deviation of 0.199, with a 95% interval of 2.212–2.942. The selected final-equation value ($\kappa=2.355$) lay within this bootstrap interval. The bootstrap-refitted test curve RMSE was ($499.99\pm37.03$), indicating moderate held-out prediction variability under resampling but no failed refitting or numerical instability.

Table S4. Fixed-structure bootstrap refitting of the denominator offset $(b_{2})$.

| Quantity | Value |
| --- | --- |
| Fixed numerator term | $softplus(2\tilde{Q})$ |
| Refitted parameter | $\kappa$ |
| Selected final-equation ($\kappa$) | 2.355 |
| Bootstrap success rate | 500/500 |
| Bootstrap ($\kappa$), mean $\pm$ SD | $2.564\pm0.199$ |
| Bootstrap ($\kappa$), median | 2.565 |
| Bootstrap ($\kappa$), 95% interval | $2.212-2.942$ |
| Selected $\kappa$ inside bootstrap interval | Yes |
| Bootstrap-refit test curve RMSE, mean $\pm$ SD | $499.99\pm37.03$ |

These results suggest that the selected symbolic structure can be refitted consistently under bootstrap resampling, while the spread of ($\kappa$) and the held-out prediction variability reflect the limited number of available formulations.
